# EXPLORING THE EVERYDAY EXPERIENCES OF FAMILY CAREGIVERS OF PEOPLE LIVING WITH DEMENTIA: A LONGITUDINAL DIARY STUDY

**DOI:** 10.1093/geroni/igad104.0338

**Published:** 2023-12-21

**Authors:** Kishore Seetharaman, Lucy Kervin, Koushambhi Basu, Jennifer Baumbusch

**Affiliations:** Simon Fraser University, Vancouver, British Columbia, Canada; Simon Fraser University, Vancouver, British Columbia, Canada; University of British Columbia, Vancouver, British Columbia, Canada; University of British Columbia, Vancouver, British Columbia, Canada

## Abstract

People living with dementia rely largely on the support of unpaid family caregivers to remain living at home in the community. The strain created by this dependence is evidenced by high rates of distress among caregivers of people living with dementia and underscores the importance of access to appropriate formal and informal supports for this group. Providing tailored and meaningful supports and services to families requires a good understanding of the multidimensional and dynamic nature of the caregiving experience. Our study employed longitudinal qualitative methodology to understand how caregivers of people living with dementia were experiencing progression of the disease and shifting support needs over a two year-period. Fourteen family caregivers in British Columbia, Canada, wrote monthly diaries reflecting on their experience of caregiving and navigating supports. Thematic analysis of 137 diary entries shed light on different facets of caregiving experience, including psychological and emotional challenges (e.g., feeling overwhelmed, lacking a sense of control, trouble maintaining sense of self) and difficulties navigating formal care and supports (e.g., lack of access to services and information, lack of flexibility in service delivery). However, accounts also reflected participants’ resilience, through a range of coping strategies adopted to resolve problems encountered in caregiving, drawing on emotional and practical support from family, friends, and community. The diaries offer critical perspectives on gaps and inadequacies in formal supports for family caregivers and opportunities to better support the health and wellness of caregivers and recipients through dementia care policy and practice.

